# Preterm labour detection by use of a biophysical marker: the uterine electrical activity

**DOI:** 10.1186/1471-2393-7-S1-S5

**Published:** 2007-06-01

**Authors:** Catherine K Marque, Jérémy Terrien, Sandy Rihana, Guy Germain

**Affiliations:** 1 Université de Technologie de Compiègne – CNRS, UMR 6600, Biomécanique et Génie Biomédical, France; 2 INRA, UMR 1198; ENVA; CNRS, FRE 2857, Biologie du Développement et Reproduction, Jouy en Josas, F-78350, France

## Abstract

**Background:**

The electrical activity of the uterine muscle is representative of uterine contractility. Its characterization may be used to detect a potential risk of preterm delivery in women, even at an early gestational stage.

**Methods:**

We have investigated the effect of the recording electrode position on the spectral content of the signal by using a mathematical model of the women's abdomen. We have then compared the simulated results to actual recordings. On signals with noise reduced with a dedicated algorithm, we have characterized the main frequency components of the signal spectrum in order to compute parameters indicative of different situations: preterm contractions resulting nonetheless in term delivery (i.e. normal contractions) and preterm contractions leading to preterm delivery (i.e. high-risk contractions). A diagnosis system permitted us to discriminate between these different categories of contractions. As the position of the placenta seems to affect the frequency content of electrical activity, we have also investigated in monkeys, with internal electrodes attached on the uterus, the effect of the placenta on the spectral content of the electrical signals.

**Results:**

In women, the best electrode position was the median vertical axis of the abdomen. The discrimination between high risk and normal contractions showed that it was possible to detect a risk of preterm labour as early as at the 27th week of pregnancy (Misclassification Rate range: 11–19.5%). Placental influence on electrical signals was evidenced in animal recordings, with higher energy content in high frequency bands, for signals recorded away from the placenta when compared to signals recorded above the placental insertion. However, we noticed, from pregnancy to labour, a similar evolution of the frequency content of the signal towards high frequencies, whatever the relative position of electrodes and placenta.

**Conclusion:**

On human recordings, this study has proved that it is possible to detect, by non-invasive abdominal recordings, a risk of preterm birth as early as the 27th week of pregnancy. On animal signals, we have evidenced that the placenta exerts a local influence on the characteristics of the electrical activity of the uterus. However, these differences have a small influence on premature delivery risk diagnosis when using proper diagnosis tools.

## Background

**Figure 1:**
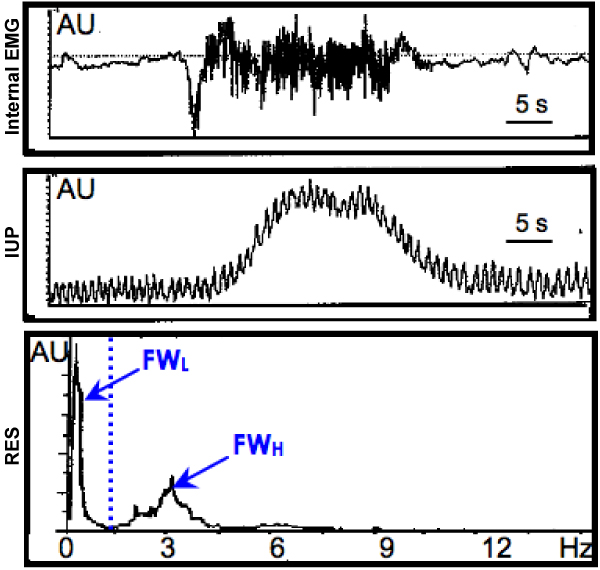
**Characteristics of the Uterine EMG**. Uterine EMG (internal electrodes) recorded on a pregnant cynomolgus monkey during pregnancy (upper trace), related Intra Uterine Pressure (middle trace) and Relative Energy Spectrum (RES) (lower trace). FW_L _and FW_H_, fast wave low and fast wave high, are the main components of the relative energy spectrum (RES), respectively. AU, arbitrary units.

The electrical activity produced by the uterus has been proved, as for other muscles, to be representative of its contractile function [1]. It has been recorded either directly with electrodes attached to the muscle in animal experiments (uterine electromyogram, EMG) [2,3] or, for obstetrical purposes, with surface electrodes placed on the abdominal skin of pregnant women (electrohysterogram EHG) [4,5]. The relationship between internal EMGs and external EHGs has been evidenced in rat and monkey experiments during pregnancy and labour [3,6]. The latter work has also permitted to define the two main components of uterine electrical activity: a high (FW_H_: fast wave high) and a low (FW_L_: fast wave low) frequency components (Figure 1). These two components have different frequency bands, depending on the species [1]. FW_L _is likely related to the propagation of the electrical activity, and FW_H _to the excitability of the uterus [1].

We have studied the possibility that the use of EHGs recorded on the abdomen of pregnant women will enable detection of contractions indicative of a high risk of preterm labour [7]. We have also recorded EMGs on the uterus during the last third of monkey pregnancy, in order to characterize their evolution along the latter part of pregnancy and to examine the influence of the placenta upon the electrical activity, according to its implantation site (anterior or posterior placental insertion). Finally, we have developed a mathematical model, in order to represent problems specific to abdominal EHG recordings, and to help interpreting the obtained results.

## Methods

### Recording abdominal EHG signals

#### Population

This study obtained the agreement of our regional ethical committee. Following informed consent, 111 recordings were made from 107 women hospitalized for risk pregnancy in the Amiens Hospital. Inclusion criteria were singleton pregnancy, term between 18 and 37 weeks of pregnancy and the presence of uterine contractions. Only one third of the women finally delivered preterm. Term labour was defined as delivery occurring after the 37^th ^week from the last menstrual period. We used bipolar Ag/AgCl surface electrodes (8 mm diameter, 2.5 cm spacing) for recording and a reference electrode was positioned on the hip of the patients. We used a device specifically developed for EHG recordings [8]. This ambulatory battery-operated device permits the 24 h recording of two EHG leads (0.05–16 Hz) and their digitization at 32 Hz sampling rate. We extracted from these recordings all the contractions, by peer review of the signals and manual segmentation based on the expertise of the operators. We then obtained 396 contractions. For the learning phase of the neural networks (see below), the contractions were distributed into learning data (257 contractions) and test data (139 contractions), to evaluate the performance of the defined networks in terms of generalization.

#### Analysis of abdominal EHG signals

**Figure 2:**
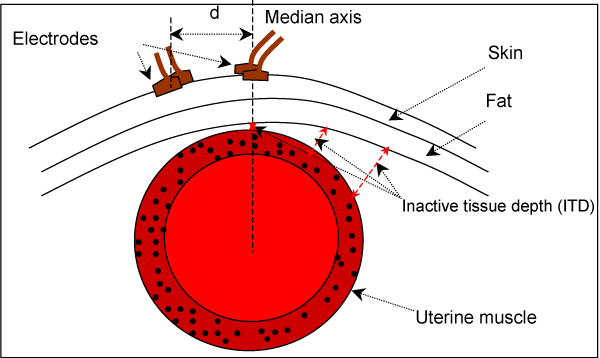
**Schematic representation of a transverse view of the pregnant abdomen in woman**. Dark red crown represents the uterine muscle. The EHG is obtained with surface electrodes placed on the abdomen permitting the recording of the EMG produced by the uterine muscle. The electrical activity propagates across different layers: a skin layer, a fat layer, and an inactive tissue layer (ITD, which is the inner content of the abdomen pushed apart by the growing uterus). The depth of the ITD layer increases as the distance d between the electrodes and the median vertical axis of the abdomen increases. ITD = 0 when the electrodes are located on the median vertical axis (d = 0).

The first requirement to monitor uterine activity is to obtain good quality signals. We have studied the influence of the position of the recording electrodes with two approaches. The first one was to use a mathematical model of the filtering effect of the tissues present between the uterus and skin electrodes (Figure 2). These tissues are associated with a transfer function that attenuates the highest frequencies of the EHG. We have tested the influence of the distance **d **between the median vertical axis of the abdomen and the electrodes, as well as the influence of the inactive tissue depth (**ITD**) of pregnant women. We have modified for this purpose the equation proposed by Farina *et al. *for the conductive volume used to model the striated muscle surface EMG [9]. We also recorded real EHG on pregnant women with a distance **d **= 5 cm and we compared these distant signals with the reference one (d = 0) by using Kruskall-Wallis and Mann-Whitney statistical tests.

Since several noises (mainly the maternal electrocardiogram, ECG) corrupt the recorded signals, and therefore modify the frequency content of raw EHG signals, we used a dedicated algorithm, previously developed in the laboratory [10], to reduce the noise of the raw EHGs. We extracted from these treated signals FW_L _and FW_H _components by using wavelet transform [11]. We computed from these components parameters representative of the two situations of interest: risk contractions (leading to preterm delivery) and normal contractions (leading to term delivery) [8].

The last step was decision making. We compared different neural networks algorithms, which are non-linear classification tools, to test the power of EHG analysis to diagnose a risk of preterm birth. These neural networks, by using the parameters extracted from the EHG bursts, permitted us to classify, for a given stage of gestation (i.e. 27–28 weeks of gestation), the contractions between two groups: i) high risk contractions: contractions recorded on women who proved to deliver preterm (i.e. at 33 weeks of gestation), ii) normal contractions: contractions recorded on women who proved to deliver at term (i.e. at term > 37 weeks of gestation). We used Multi Layer Perceptrons with one hidden layer and supervised learning. Cross-validation with a classified subset was used for the learning phase. We tested two different learning algorithms. The first one is based on the gradient back-propagation with momentum (BPM) [12] and the second one uses the Levenberg-Marquardt algorithm (LM) [13]. The latest algorithm is associated with Bayesian regularization in order to reduce over-fitting during the learning phase [14]. The results given by these classification tools were compared to the real term at delivery, permitting thus to compute the misclassification rate, specificity, sensitivity and negative predictive value.

### Recording internal EMG signals

Internal EMG signals were obtained from three pregnant cynomolgus monkeys by the implantation of a telemetric transducer TL 11M3-D70-CCP (Data Sciences International, St. Paul, MN, USA) having specific filters (1st order high-pass Bessel filter (0.1 Hz), 5th order low-pass Bessel filter (100 Hz)). The transducer contains two bipolar EMG leads, which were sutured on the uterine wall, and one pressure catheter, which was used to measure the intra uterine pressure (IUP). The transducer was implanted chronically at 120 days of gestation and remained until labour. We sutured one EMG bipolar lead above the placenta (P) and the other away from the placental implantation site (NP). Our Institutional Animal Use and Care of Laboratory Research Animal Committee approved the procedures. Signals were digitized with a 50 Hz sampling rate. Contractions were extracted off line by a peer review of the signals and distributed according to three gestation stages (namely gestation not in labour, pre-labour phase and labour phase), by analysing the number of contractions per 10 minutes, and the amplitude of the intra-uterine pressure. For each contraction, we calculated the relative energy spectrum (RES) of the associated EMG.

## Results

### Abdominal EHG signals

**Figure 3:**
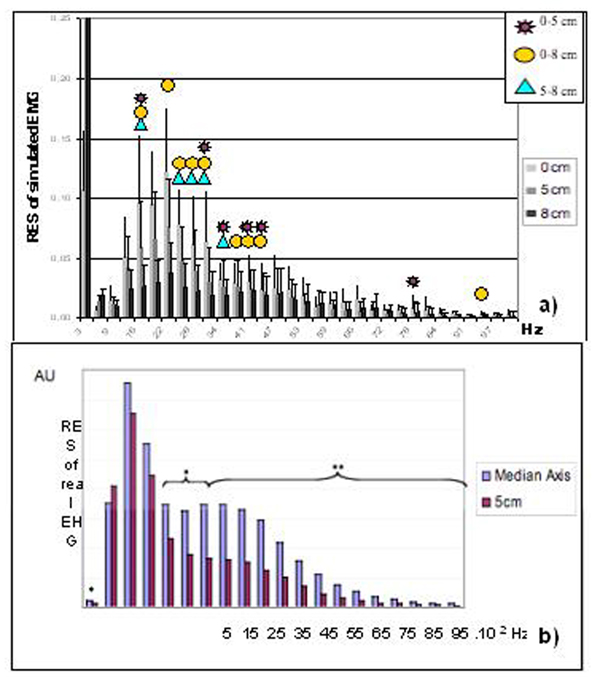
**Analysis of electrode position on the relative energy spectrum of simulated and real uterine electromyograms**. **(a) **Relative energy spectrum (RES) computed from simulated signals with three bipolar-electrode positions (see figure 2): **d **= 0 cm (Pair1), **d **= 5 cm (Pair2) and **d **= 8 cm (Pair3). The lowest frequency band has been truncated for a better visualisation of the other frequency bands. Significant differences resulting from the electrode positions are indicated by stars, circles and triangles, (p < 0.05). **(b) **RES computed from EHG recorded in pregnant women with two electrode positions: **d **= 0 cm (Median Axis) and **d **= 5 cm. (*p < 0.05; **p < 0.01). AU, arbitrary units.

The mathematical modelling of the electrode position effect has investigated **d **and **ITD **influences separately and then both influences together. **ITD **influences the very high frequency content of the EHG spectrum and **d **influences the median and high part of the spectrum (Figure 3a). Both effects create a strong attenuation of the signal energy.

In real signals, **d **and **ITD **effects are linked because **ITD **increases when the distance **d **between the electrodes and the median vertical axis of the abdomen, increases. For real signals recorded with different electrode positions, we can notice in the median part of the spectrum a strong influence of the electrode position. The main effect seems therefore to be related to **d **increase (Figure 3b).

We have used signals recorded on the median vertical axis to examine the possibility of discrimination, for a given stage of pregnancy at recording, of contractions recorded on women who subsequently delivered before term and contractions recorded on women who subsequently delivered at term. The result of the classification is given in terms of misclassification rate (MR) (Table 1) and of sensibility (Se), specificity (Sp) and negative predictive value (NPV) (Table 2). The misclassification is lower with the LM algorithm than with the BPM algorithm. This is also true for the number of unclassified contractions. They range from 3–45% for BPM, compared to less than 2.5% for LM (data not shown). However, if we compare the Se, Sp and NPV for both network algorithms, the results are better for the BPM algorithm, whatever the stage of pregnancy is (Table 2).

### Internal EMG signals

**Figure 4:**
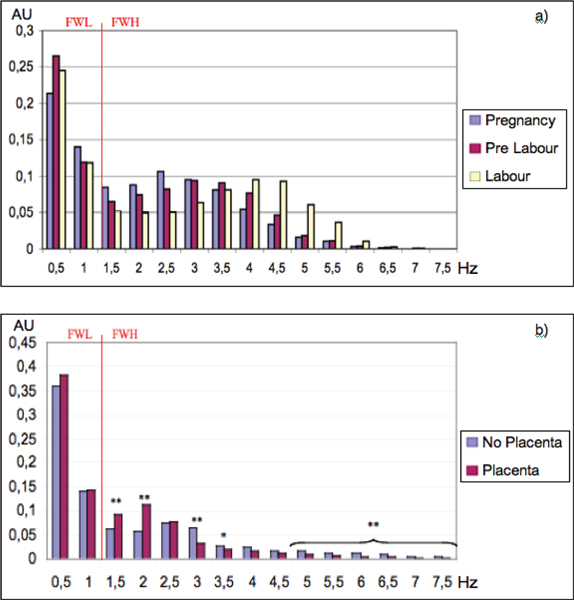
**Analysis of term and placental influences on the relative energy spectrum of uterine electrical signals**. **(a) **Relative energy spectrum (RES) computed from pregnant monkey EMGs recorded during the last third of pregnancy and labour. Notice the shift towards higher frequencies as pregnancy progresses. **(b) **RES computed from pregnant monkey EMG's recorded during pregnancy, with two electrode positions: electrodes away from the site of placenta insertion (No Placenta); electrodes located above the placental insertion (Placenta) (**p < 0.01). AU, arbitrary units.

Figure 4a presents the result of the evolution of the internal EMG spectral content recorded on the monkey uterus during pregnancy. We notice on RES a global shift of the spectral content towards high frequencies, from pregnancy, not in labour, to the stage of labour. As pregnancy progresses, the relative energy of FW_H _increases, subsequently associated with a decrease in FW_L _relative energy.

Figure 4b presents the placental influence on the RES of EMG recorded during pregnancy not in labour. The main placental influence is a shift of FW_H _towards lower frequencies when the electrodes are located above the placental (P) insertion. However, we evidenced the same global shift of the content of the EMG signal towards higher frequencies, as pregnancy progresses, whatever the electrode position was with respect to the placenta insertion.

Furthermore, if we study the placental influence on EMG associated with labour contractions, the differences between "No Placenta" and "Placenta" EMG RES is decreased (data not shown).

## Discussion

The first step to monitor biomedical signals is to record them in the best conditions. For the EHGs recorded on women, our study proved that the best way to position the electrodes, as far as the frequency content of the EHG is concerned, is to align the electrode pairs with the median vertical axis of the uterus. In this location, the electrodes are closer to the uterus and the spectral content of the EHG presents higher energy in the FW_H _frequency band.

Concerning the diagnosis of risk contractions, a problem remains for the still limited pertinence of our results because at best, the results of the LM algorithm ranges are: MR = 11%–19.5%, Se = 0.79–0.82, Sp = 0.87–0.88, NPV = 0.85–0.87. One explanation is the low number of contractions available for learning, compared to the number of parameters that were fed into the learning system. In line with our previous results [15], we chose in this study, to divide the data between subclasses belonging to coherent physiological situations (weeks of pregnancy, influence of the placenta). Our animal study evidences an effect of the stage of gestation and of the placenta on the spectral content of the uterine EMG. We can conclude from both human and animal studies, that it is no longer necessary to distinguish the specific influence of the placenta. Indeed, even if we have observed differences between signals recorded at placental and extra-placental sites, the signals evolve the same way during pregnancy. During labour, their differences decrease even more. This could explain why by using efficient diagnosis tool (i.e. LM algorithm), we did not note different results for placental and extra-placental signals, as noticed in previous studies by using linear discrimination tools [7,8]. The next step of the analysis will be to reduce again the number of parameters used at the input of the network by using, for example, methods based on wavelet networks [16].

The animal study has allowed us to show evidence for the changes along pregnancy, of the spectral uterine EMG content. The shift of the uterine EMG spectrum towards higher frequencies, when the date of the term approaches, has already been mentioned in previous experiments [2-4]. We report it here for the first time in the primate uterus, based on a true longitudinal analysis. The high frequency content of EHG (FW_H_) seems therefore to be of great importance to characterise contraction efficiency, thereby justifying the need for efficient recording and filtering techniques [10] of uterine electrical signals.

## Conclusion

We have developed suitable signal processing tools to characterize the uterine contractions detected by their electrical activity during pregnancy, in monkeys and in the human. On animal signals, we have shown that the placenta exerts a local influence on the characteristics of the EMG, which changes along pregnancy. Proper diagnosis tools permit us to elucidate the diagnosis bias of the placental influence on the signals. On human recordings, we have proved that it is possible to detect the risk of preterm birth (identification of high risk contractions) by using EHG processing, at stages as early as at 27 weeks of pregnancy, and therefore to make an early diagnosis of preterm labour from the analysis of abdominal EHG, irrespective of where the placental insertion is. Collectively, these works allowed us to determine a reliable procedure to record properly the electrical uterine activity from the abdomen of pregnant women.

## Abbreviations

BPM, back propagation with momentum algorithm; EHG, electrohysterograms; EMG, electromyograms; ITD, inactive tissue depth; LM, Levenberg-Marquardt algorithm; MR, Misclassification Rate; NP, extra-placental site of the uterus; NPV, Negative Predictive Value; P, placental site of the uterus; RES, relative energy spectrum; Se, Sensitivity; Sp, Specificity.

## Competing interests

The authors declare that they have no competing interests.

## Authors' contributions

CM has been involved in the conception and design of the recording protocols, in the analysis and interpretation of data as well as in drafting the manuscript. JT has been involved in the conception and design of the recording protocols, in human and animal signal recordings, in the design of software for analysis and interpretation of data. He also revised the manuscript. SR has been involved in the development of the mathematical model as well as for analysis and interpretation of data. She also revised the manuscript. GG has been involved in the conception and design of the animal recording protocol, in the analysis and interpretation of data as well as in revising the manuscript. All the authors have given final approval of the version to be published.

## Figures and Tables

**Table 1:** Neural network misclassification rates (MR) for the prediction of risk contractions (whole population, learning data + test data)

**Weeks of pregnancy**	**27–28**		**29–32**		**33–37**	
**Placenta insertion**	**Posterior**	**Anterior**	**Posterior**	**Anterior**	**Posterior**	**Anterior**

**BPM algorithm**	18%	na	76%	26%	19%	19%
**LM algorithm**	13%	na	19.5%	18%	12%	11%

BPM, back propagation with momentum algorithm; LM, Levenberg-Marquardt algorithm; na, data not available (too few number of recorded contractions).

**Table 2:** Diagnosis performances of neural network algorithms for the prediction of preterm delivery

**Algorithm**	**BPM**		**LM**	
**Placenta insertion**	**Posterior**	**Anterior**	**Posterior**	**Anterior**

**Sensitivity (Se)**	0.85	0.90	0.82	0.79
**Specificity (Sp)**	0.93	0.91	0.88	0.87
**Negative predictive value (NPV)**	0.88	0.92	0.87	0.85

BPM, back propagation with momentum algorithm; LM, Levenberg-Marquardt algorithm.
